# Supplementary material for: Using artificial neural networks to reveal the human confidence computation
Source: PLoS Comput Biol. 2025 Dec 29;21(12):e1013827. doi: 10.1371/journal.pcbi.1013827 (PMC12799183; doi:10.1371/journal.pcbi.1013827)
Supplement: S1 Text. — (DOCX) [file pcbi.1013827.s001.docx]

**Supplementary Information**

**Using artificial neural networks to reveal the human confidence computation**

Medha Shekhar, Herrick Fung, Krish Saxena, Farshad Rafiei and Dobromir Rahnev

**Supplementary methods**

We performed model comparisons as in the main paper but using three other CNN architectures – MSDNet (multi-scale dense network; Huang et al., 2017), BLNet (a recurrent convolutional network; Spoerer et al., 2020) and CNet (a parallel cascaded network; Iuzzolino et al., 2021) – that were previously trained on MNIST and fit to human choice and RT data (Rafiei et al., 2024). We reasoned that testing across architectures would enable us to understand how well other CNNs architectures can fit human data relative to RTNet and whether the same confidence strategies are consistently supported across model architectures. All the networks were implemented in Python (version 3.10.11). We briefly describe the network architectures below.

CNet

The Cascaded parallel network (CNet) uses parallel processing and introduces propagation delays between convolutional layers using skip connections (Iuzzolino et al., 2021). Even though all the network’s layers process input parallelly at any given time, propagation delays cause early layers to receive input faster and achieve stable activations sooner. For the same amount of processing time, the later layers receive only partial input from the earlier layers and take more processing steps to achieve stabilization of their output. As a result, simple features propagate faster through the network while more complex features require greater processing time, naturally leading to the trade-off between processing speed and stimulus complexity found in humans. The network’s decision can be generated by setting a threshold at the output layer and the decision time is determined by the number of processing steps that are required to reach the threshold. The network was implemented within the ResNet-18 architecture since it requires network architectures with skip connections (He et al., 2015). ResNet-18 contains 17 convolutional layers organized in eight residual blocks and one fully connected output layer with a softmax activation function that generates the decision.

BLNet

BLNet is a recurrent convolutional neural network (RCNN) that uses bottom-up and lateral connections to recurrently feed each layer’s input back to itself (Spoerer et al., 2020). Therefore, each layer receives feedforward input from the previous convolutional layer as well as recurrent input from itself in the form of its own activations at the previous time step. Time steps are defined in terms of feedforward sweeps and after each feedforward sweep, the network’s readout is evaluated. If the readout crosses a predefined threshold, the network chooses the option with the highest readout. The number of feedforward sweeps preceding the decision determines the response time. Recurrent processing is a biologically inspired mechanism that can dynamically adjust a network’s computational power. For instance, setting a higher threshold will lead to the network undergoing a larger number of feedforward and recurrent computations, effectively resulting in a deeper network being unrolled. We implemented BLNet as described in the original publication that introduced this network (Spoerer et al., 2020), which consists of seven convolutional layers and a final readout layer with a softmax activation function. The network was unrolled across time for a maximum of eight time steps.

MSDNet

MSDNet uses a standard feedforward CNN with early-exit classifiers after each convolutional layer (Huang et al., 2017). The classifiers compute the evidence at each layer using a softmax function and if the evidence crosses a pre-defined threshold, the network stops processing input and generates a response based on that layer’s output. The decision corresponds to the choice option that generates the highest softmax value and the response time corresponds to the layer at which the decision was generated. As in the original publication, MSDNet was implemented with the AlexNet architecture with five convolutional layers and three fully connected layers.

Fitting to confidence data

We followed the same approach as for RTNet to fit the networks to human confidence. We trained 60 instances of each network using different random initializations of the networks’ weights to allow for individual differences in learning. We then fit them to human choice data by optimizing the noise and threshold parameters to provide the closest match to human accuracy. For CNet, the closest match was achieved for noise levels of 1.42 for easy images and 1.83 for difficult images and threshold values of 0.83 for the speed condition and 0.9 for the accuracy condition and. For BLNet, the best match to human accuracy was obtained when the noise levels were set to 0.55 for easy images and 1.2 for difficult images and when thresholds were set to 0.4 for the speed condition and 0.95 for the accuracy focus condition. For MSDNet, the closest match was achieved for noise levels of 1.9 for easy images and 3.0 for difficult images and for threshold values of 0.58 for the speed condition and 0.82 for the accuracy condition.

We then fit subject-specific confidence criteria to these models to obtain confidence on a 4-point scale. We generated confidence using four confidence strategies – PE, Top2Diff, Softmax and Entropy (computed using Softmax probabilities) and fit the raw confidence values to each individual subject to generate the models’ confidence ratings on a 4-point scale. Comparing the fits from these four strategies across the four network architectures showed that the RTNet architecture gave the closest fits to human choices and confidence, substantially outperforming all the other architectures by at least 430 AIC points (see Supplementary Figure 1). Therefore, it is unlikely that extensively comparing confidence strategies within these architectures will be very informative, especially given that all probability-based models are computationally expensive to fit. Instead, we decided to mainly focus on comparing the seven different kinds of confidence strategies only within the best performing architecture – RTNet.

**References**

1. He, K., Zhang, X., Ren, S., & Sun, J. (2015). Deep Residual Learning for Image Recognition. *Proceedings of the IEEE Computer Society Conference on Computer Vision and Pattern Recognition*, *2016-December*, 770–778. https://doi.org/10.1109/CVPR.2016.90
2. Huang, G., Chen, D., Li, T., Wu, F., Van Der Maaten, L., & Weinberger, K. (2017). Multi-Scale Dense Networks for Resource Efficient Image Classification. *6th International Conference on Learning Representations, ICLR 2018 - Conference Track Proceedings*. https://arxiv.org/abs/1703.09844v5
3. Iuzzolino, M. L., Mozer, M. C., & Bengio, S. (2021). Improving Anytime Prediction with Parallel Cascaded Networks and a Temporal-Difference Loss. *Advances in Neural Information Processing Systems*, *33*, 27631–27644. https://arxiv.org/abs/2102.09808v4
4. Rafiei, F., Shekhar, M., & Rahnev, D. (2024). The neural network RTNet exhibits the signatures of human perceptual decision-making. *Nature Human Behaviour 2024*, 1–19. https://doi.org/10.1038/s41562-024-01914-8
5. Spoerer, C. J., Kietzmann, T. C., Mehrer, J., Charest, I., & Kriegeskorte, N. (2020). Recurrent neural networks can explain flexible trading of speed and accuracy in biological vision. *PLoS Computational Biology*, *16*(10). https://doi.org/10.1371/JOURNAL.PCBI.1008215
