# Supplementary figures and images for: Using artificial neural networks to reveal the human confidence computation
Source: PLoS Comput Biol. 2025 Dec 29;21(12):e1013827. doi: 10.1371/journal.pcbi.1013827 (PMC12799183; doi:10.1371/journal.pcbi.1013827)

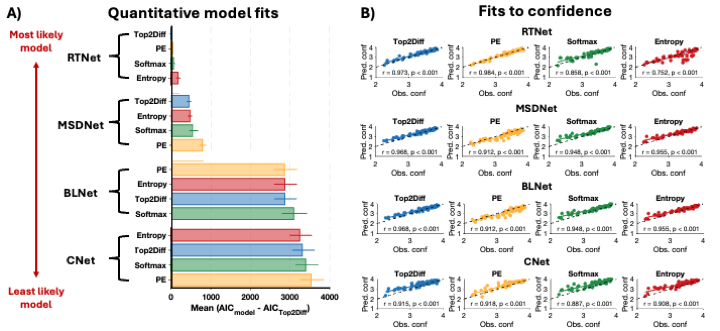

Supplement: S1 Fig — (A) Quantitative comparisons using AIC scores. Positive AIC differences indicate support for the RTNet-Top2Diff model. We first assessed the models’ fits to the data by computing average AIC values across the 60 subjects for the 16 models (4 architectures x 4 confidence strategies). We found that the confidence strategies built on top of RTNet generated substantially lower AIC values compared to the remaining 12 models (3 architectures x 4 confidence strategies). The Top2Diff strategy instantiated within RTNet remained the best model, with other strategies instantiated within RTNet falling between 45–170 AIC points behind it. In contrast, the four confidence strategies instantiated within MSDNet were 430–778 points worse than the RTNet-Top2Diff model. BLNet and CNet produced even worse fits with strategies instantiated in BLNet falling behind the RTNet-Top2Diff model by 2860–3087 AIC points and strategies within CNet falling behind RTNet-Top2 Diff by 3245–3528 AIC points. All AIC comparisons were significant as assessed by computing bootstrapped 95% confidence intervals. These results corroborate our previous findings that RTNet provides the best fits to human data [28]. (B) The poor fits to data by other CNN architectures suggest that these architectures are unable to simultaneously fit the observed patterns of choices and confidence. However, since fitting models to choices and confidence was done in separate steps, it is important to rule out the possibility that these poor fits are due to a failure of models to fit confidence alone. Therefore, we correlated each subject’s average confidence to the model’s average confidence for that subject. We found that all models and strategies produced very high correlations (median r = 0.934, all r’s > 0.75, all p’s < 0.001), confirming that the fitting procedure was successful. Instead, the poor fits for MSDNet, BLNet, and CNet were likely a result of the failure of these architectures to fit the pattern of human choices u [file pcbi.1013827.s002.tiff]
