# Supplementary material for: Using artificial neural networks to reveal the human confidence computation
Source: PLoS Comput Biol. 2025 Dec 29;21(12):e1013827. doi: 10.1371/journal.pcbi.1013827 (PMC12799183; doi:10.1371/journal.pcbi.1013827)
Supplement: Attachment — Submitted filename: Rebuttal.docx [file pcbi.1013827.s004.docx]

Dear Dr. Doumas and Dr. Graham,

Thank you for giving us the opportunity to revise and improve our manuscript. We also want to thank all the reviewers for their helpful comments and suggestions. Firstly, based on concerns from Reviewer 2 regarding the novelty of our findings, we have now added three new confidence models testing three different hypotheses regarding how confidence is computed – 1) confidence as the posterior probability of the choice (the Bayesian confidence hypothesis; Aitchison et al., 2015), 2) confidence as a difference in posterior probability between the top-two choices (Li & Ma, 2020), and 3) confidence as a difference in probability between the choice and the average across the remaining choices (Comay et al., 2023). Adding these models allows us to make novel conclusions since these models have never been systematically compared before. Particularly, with these results, we can now inform the debate on whether confidence relies on probability computations or raw evidence.

Secondly, we have addressed concerns from Reviewer 1 that our model prediction analyses do not take into account the accuracy of the choice underlying the confidence ratings. As the reviewer points out, this can sometimes lead to comparisons that are not meaningful. To resolve this issue, we have replaced the image-by-image analysis of confidence predictions to a stimulus category-wise analysis of predictions, which allows us to account for choice accuracy.

Even when compared to these additional models, we find that the Top2Diff model (which computes confidence as the difference in raw evidence between the top-two choices) provides the best quantitative fits. These findings challenge previous findings by Li & Ma (2020) and Comay et. al (2023) by showing that confidence derived as the difference in evidence between the top-two choices is sufficient to explain human data. These results also suggest that confidence is more likely to rely on simple computations involving raw evidence rather than complex probability calculations. We believe our paper is much improved as a result of these additions. We provide a point-by-point response to the reviewers’ comments below.

Sincerely,

Medha Shekhar, Herrick Fung, Krish Saxena, Farshad Rafiei and Dobromir Rahnev

**Reviewer #1**

***This paper makes a clear case for a simple point: comparing top 2 options gives the best prediction of confidence ratings. The authors use multiple different analyses to support this point, including individual and group comparisons and exploring other network architectures. The claim is also consistent with cited past work.

What was lacking in the paper was an analysis of the choices made by the models. Specifically, are these confidence ratings taken only from images where the subject and the model make the same choice? It is hard to think about how to compare the model's confidence in an incorrect choice to a subject's*** ***confidence in their correct choice or vice versa.***

We thank the reviewer for catching this issue with our analysis. In the image-by-image prediction analysis, for each image, we obtain the average confidence across all subjects and correlate it with the model’s average confidence for that image across all ANN instances. This analysis can sometimes result in rewarding models whose confidence is not predictive of accuracy.

For the image-by-image prediction analysis, it is difficult to separate correct and incorrect choices since it would lead to the exclusion of varying proportion of subjects for every image, resulting in each image-by-image confidence estimate having a different reliability. Therefore, we decided to modify our analyses to compare how well models can predict stimulus category-wise confidence. For each subject, we can compute the average confidence for each stimulus category separately for correct and incorrect trials which can then be correlated with the corresponding quantity in the models.

These analyses revealed systematic differences in how well models can fit variations in confidence across stimulus categories for error trials. In fact, the models that produced the worst AIC fits uniformly over-estimated confidence for error trials – both on average and within individual subjects. Indeed, the good AIC fits of the Top2Diff model can be explained by its ability to fit confidence for both correct and error trials as well as the patterns of variations in confidence across different stimuli. We paste the results of these analyses below (note that in the new version of the paper, we include 3 more models: the Bayesian Confidence Hypothesis (BCH) model, ProbTop2Diff model from Li & Ma, 2020, and ProbAvgRes model from Comay et al., 2013):

Assessing model predictions of confidence for stimulus categories

Apart from capturing the behavioral patterns of different experimental manipulations, a robust model of confidence should also account for stimulus-driven variations. Specifically, certain categories of stimuli (such as the number 1) may be more prone to confusion with other digits (such as 7) and therefore, may be associated with lower confidence on average. Therefore, we assessed whether models could mimic the observed patterns of confidence across the eight stimulus categories. In addition to stimulus categories, we separated trials based on choice accuracy, since observed patterns of confidence depend on the accuracy of the primary choice. Confidence on error trials is generally lower compared to correct trials and confidence for correct and error trials have opposing relationships with stimulus discriminability (**Figure 3C**). Mixing confidence across correct and error trials can thus obscure the underlying patterns.

First, we computed average confidence as a function of choice accuracy and stimulus category. Although confidence was generally well matched across the eight stimulus categories, there were small variations between categories. For correct trials, humans tended of give the highest confidence for the digit 8, and the lowest confidence for the digit 1. For error trials, the digit 8 was again associated with the highest confidence while 6 was associated with the lowest confidence. As expected, confidence was always higher for correct trials compared to error trials for humans as well as all models across all stimulus categories (**Figure 4**). Model comparisons showed that the Top2Diff model gave the closest fits to the overall pattern of confidence as assessed by the sum of squared errors (Top2Diff = 1.391; ProbAvgRes = 2.100; ProbTop2Diff = 2.035; Entropy = 2.427; PE = 3.402; Softmax = 2.088; BCH = 2.231). These errors were significantly lower for the Top2Diff model compared to all other models (ProbAvgRes: t(59) = 7.970, p < .001; ProbTop2Diff: t(59) = 6.124, p < 0.001; Entropy: t(59) = 4.141, p < 0.001; PE: t(59) = 9.922, p < 0.001; Softmax: t(59) = 3.169, p = 0.002; BCH: t(59) = 6.780, p < 0 .001). In particular, for error trials, all other models produce substantial deviations from observed confidence for at least two stimulus categories, with the PE, Softmax, and Entropy models consistently overpredicting confidence across all stimulus categories.

***Figure 4. Stimulus category-wise predictions of average confidence for correct and error trials.*** *Confidence was uniformly greater for correct trials compared to error trials across all eight stimulus categories. In addition, there were small variations in confidence across the eight categories, possibly due to some digits being more prone to confusion with others. All confidence strategies correctly predicted that confidence was higher for correct trials compared to error trials, but Top2Diff best captured the variations in confidence found between the stimulus categories. In particular, all models except Top2Diff over-estimated confidence for error trials for at least two categories, with the PE, Softmax, and Entropy models over-estimating confidence for all stimulus categories. Error bars depict SEM. SSE, sum of squared errors (smaller values indicate better fits).*

Second, we analyzed how well models predicted the variations in confidence across stimulus categories for individual subjects. For each subject, we plotted averaged confidence within each stimulus category against the model’s predicted confidence for that stimulus category, separately for correct and error trials. For correct trials (**Figure 5A**), all confidence strategies were able to predict individual confidence within the stimulus categories well – with predicted confidence falling within the observed range and showing a continuous increase within the range. None of the models showed a substantial tendency to over- or under-estimate confidence. However, for error trials (**Figure 6A**) the Entropy, PE, and Softmax models uniformly over-estimated confidence across stimulus categories as well as individuals, which may explain their poor fits, compared to the top-three models – Top2Diff, ProbAvgRes and ProbTop2Diff.

***Figure 5. Stimulus category-wise model predictions of individual confidence for correct choices.*** *(A) We computed average confidence for correct trials within each stimulus category separately for each individual subject and plotted these against the corresponding quantities predicted by each model. All models generated strong and significant correlations with observed confidence, suggesting that they were able to capture stimulus-related variations in confidence. (B) The Top2Diff strategy generated the numerically highest correlations with human confidence. However, these correlations were not significantly higher than those generated by ProbAvgRes, ProbTop2Diff, Entropy, and PE models. Error bars show 95% confidence intervals. (C) We fit a linear-mixed model that quantified how closely each model captured observed category-wise variations in confidence, while controlling for the repeated measurement across individuals. AIC scores derived from the linear mixed models showed that the Top2Diff strategy generated the best fits to category-specific confidence for correct trials with an AIC difference of at least 10 points with all other strategies.*

***Figure 6. Stimulus category-wise model predictions of individual confidence for error trials.*** *(A) We computed average confidence for error trials within each stimulus category separately for each individual subject and plotted these against the corresponding quantities predicted by each model. As with correct trials, models generated strong and significant correlations with observed confidence, suggesting that they are generally able to capture stimulus-related variations in confidence. (B) The Top2Diff strategy generated the numerically highest correlations with human confidence, but these correlations were not significantly higher than those generated by ProbAvgRes, ProbTop2Diff, PE and BCH models. Error bars show 95% confidence intervals. (C) We fit a linear-mixed model that quantified how closely each model captured observed category-wise variations in confidence, while controlling for the repeated measurement across individuals. AIC scores from these regression models showed that the Top2Diff strategy generated the substantially better fits to category-specific confidence for error trials with an AIC difference of at least 62 points with all other strategies.*

To quantify how well models could predict the variations in confidence between stimulus categories, we fit a linear mixed model which predicted human confidence for each stimulus category from the model’s confidence for that category. Since the category-wise predictions of confidence were obtained separately for each individual subject, we controlled for the repeated measurements of these stimuli by including stimulus category as a random effect in the model. Using the mixed linear model’s estimates of slope and intercept, we generated the model’s predictions of category-wise confidence across individuals and correlated them with their corresponding observed quantities.

For correct trials, all strategies produced very strong correlations with human confidence (all r’s > 0.871, all p’s < 0.001; **Figure 5B**) across the eight stimulus categories. Comparing the Pearson’s r-values showed that the Top2Diff model generated the numerically highest correlations, which were significantly higher than those produced by the Softmax and BCH models (Softmax: z(479) = 3.789, p < 0.001; BCH: z(479) = 3.329, p < 0.001). However, the Top2Diff model’s correlations were not significantly higher than the ProbAvgRes, ProbTop2Diff, Entropy, and PE models (ProbAvgRes: z(479) = .939, p = 0.348; ProbTop2Diff: z(479) = .196, p = .844; Entropy: z(479) = .196, p = .844; PE: z(479) = .576, p = .565). In addition to these correlations, we also quantified the goodness-of-fit of the linear mixed model using AIC scores and found that the Top2Diff strategy generated the closest predictions of category-wise confidence for correct trials across individual subjects, with a difference of at least 10 AIC points with all other strategies (**Figure 5C**).

For error trials, the category-wise correlations between model and human confidence were slightly lower but nevertheless strong and significant for all confidence strategies (all r’s > 0.818, all p’s < 0.001; **Figure 6B**). Comparing the Pearson’s r-values showed that the Top2Diff model once again generated the numerically highest correlations, which were significantly higher than those produced by the Entropy and Softmax models (Entropy: z(479) = 2.100, p = 0.036; Softmax: z(479) = 2.627, p = 0.009). However, the Top2Diff model’s correlations were not significantly higher than those produced by the ProbAvgRes, ProbTop2Diff, PE, and BCH models (ProbAvgRes: z(479) = 1.540, p = .124; ProbTop2Diff: z(479) = 1.644, p = .100; PE: z(479) = .1.110, p = .267; BCH: z(479) = .944, p = .345). Comparison of AIC scores from the linear mixed model showed that the Top2Diff strategy also generated the best fits to category-wise confidence for error trials across individual subjects, with a substantial difference of at least 62 AIC points with all other strategies (**Figure 6C**). Together, these results suggest that the Top2Diff strategy best captures the patterns of variations in human confidence arising from dependencies in the stimulus categories.

***Furthermore, do the different architectures tested have better or worse correspondence to human choice on an image-by-image basis? This could be relevant for understanding their confidence rating differences (this is hinted at in the results but I don't think it was reported on directly).***

In previous work, where we developed and tested the RTNet model, we indeed compared our model with other architectures in terms of their ability to predict human choices and RTs on an image-by-image basis (Rafiei, Shekhar & Rahnev, 2024, Nat Hum Beh). RTNet showed the highest image-by-image correlations with human RT, choice as well as confidence, compared to all other architectures. In fact, because the other architectures are clearly inferior in fitting the human choices, we have decided to move the results for those architectures to the Supplementary. We made this decision in part due to the fact that we have now added three more confidence models, and felt that it’s best to focus the main paper on the distinctions between all of these confidence models without introducing architectures that are anyway not fitting well. We now include this clarification in the main text and paste the relevant section below:

For completeness, we performed similar model comparisons using three other CNN architectures – MSDNet (multi-scale dense network; Huang et al., 2017), BLNet (a recurrent convolutional network; Spoerer et al., 2020) and CNet (a parallel cascaded network; Iuzzolino et al., 2021) – that were previously trained on MNIST and fit to human choice data (Rafiei et al., 2024). These networks provide relatively poor fits to the human choice and RT data (Rafiei et al., 2024), making any confidence model comparisons built on them less informative. Nevertheless, we report confidence model comparisons within these architectures in the Supplementary Methods, Supplementary Figures 1 and 2 and Supplementary Table 1.

***Do the authors think that performing step 2 training on individual data would impact the results in any way?***

In step 2 of the model fitting procedure, we estimate the model’s parameters for generating the choices. Instead of fitting the parameters to each individual subject, we estimate these parameters at the group level by fitting the average accuracy of the model to the average accuracy of subjects, separately for each noise and speed-accuracy trade-off (SAT) condition. The reason we fit the models in this manner is because during the parameter search, RTNet is required to be simulated over 60 instances for each parameter the search algorithm chooses. Since RTNet is a very large model with millions of weights, each simulation costs substantial computational resources and time. As of now, it is not practical to fit RTNet individually to human subjects using traditional methods. However, even though model fitting was done at the group level, RTNet’s choices, RTs and confidence correlated significantly with those of individual subjects. Further, since all the confidence strategies were fit for the same model of choice, we don’t expect that this method will significantly impact the results. Nevertheless, we acknowledge this limitation in our discussion and paste the relevant section below:

One limitation of our study was the inability to fit the neural network models directly to choice and confidence data for individual subjects. Since neural networks have parameters in the order of millions, it becomes computationally intractable to simultaneously tune all these parameters and fit the observed human data. Therefore, we used a general-purpose training method to teach the models to perform the task and then optimized two critical parameters that controlled the models’ performance levels. Even then, we fit these two parameters to the average human data (instead of each subject separately) because RTNet requires significant computational resources and processing time to generate responses even on a single trial. Instead, the only parameters we could fit on a subject-by-subject basis using traditional exhaustive search that is standard in cognitive models (Acerbi & Ma, 2017; Nelder & Mead, 1965) were the three confidence criteria. Since all the confidence strategies were fit for the same model of choice, these limitations are not expected to significantly impact the results we report here. Future work should focus on developing methods that would allow traditional maximum likelihood estimation to be applied so that neural network models can fit individual subjects.

***The authors find no confidence difference between speed-focused and accuracy-focused trials. Can they remark on if this is consistent with prior studies?***

The effects of manipulations of speed and accuracy focus tend to depend strongly on the details of how those manipulations were introduced and the complexity of the task. In our case, we had a rather complex task (8 possible choices) that seems to have made the effect of the SAT manipulation relatively small. Indeed, the SAT manipulation had a very small effect on accuracy (2-3%; significant for the easy stimuli but not for the difficult stimuli), making it less surprising that the effect on confidence was also very small (and not significant). We checked the literature and found that two previous studies (across three different experiments) reported an increase in confidence from the speed-focus to the accuracy-focus conditions (Pleskac & Busemeyer, 2010; Rahnev et al., 2016), suggesting that increases in confidence do indeed occur with SAT manipulations. In our study, it is likely that we did not find a significant increase in confidence from the speed to accuracy-focused conditions due to a small effect size. Due to the weak overall difference in accuracy between the speed and accuracy conditions, the overall change in confidence from the speed to accuracy-focus conditions may not have reached significance. We now discuss these results in more detail in the Discussion and paste the relevant section below:

A final limitation of our study is that the complexity of the task may have made it difficult to realize the speed-accuracy trade-off manipulation. Our task structure was relatively complex – featuring eight choice options and a 2 x 2 factorial design with difficulty and SAT manipulations. Such a complex task structure may have reduced the effect of the SAT manipulation. Indeed, the SAT manipulation had a very small effect on accuracy (2-3%; significant for the easy stimuli but not for the difficult stimuli), making it less surprising that the effect on confidence was also very small (and not significant). Previous studies, on the other hand, have reported an increase in confidence from the speed-focus to the accuracy-focus conditions (Pleskac & Busemeyer, 2010; Rahnev et al., 2016), suggesting that increases in confidence do indeed occur with SAT manipulations. All confidence strategies examined here led to an increase of confidence from the speed-focus to the accuracy-focus conditions. Given the considerations above, we believe that this divergence from the current data should not be seen as a limitation of the models but as a limitation of the current dataset.

***"However, while this type of computation may not match well what humans do, it must be
noted that it produces more informative confidence ratings." What does this mean and what is it based on?***

We apologize for the confusion. We mean that computations such as Entropy and Softmax compute confidence from all the available information – i.e., using the entire distribution of evidence across all choices – whereas strategies such as Top2Diff and PE only use a subset of the available information. As a result, Entropy and Softmax contain more information about the primary decision than PE and Top2Diff. However, these strategies do not fit human data as well as Top2Diff, suggesting that humans don’t base their confidence on computations such as Entropy and Softmax. We now clarify this point and paste the relevant section below:

The standard practice in neural network modeling is to derive confidence using the Softmax (and less often the Entropy) computation. Our results demonstrate that this way of deriving confidence may not match how humans generate confidence. Nevertheless, these strategies likely produce more informative confidence ratings because they utilize all the information available from the primary decision. Therefore, Softmax and Entropy may be better suited to derive confidence from neural networks when the goal is to maximize insight into the network’s performance rather than model human behavior.

***Do the authors have anything to note about what aspects of the data that the models still don't match, in order to inspire future research?***

In our previous work (Rafiei, Shekhar, & Rahnev, 2024), we extensively analysed RTNet’s ability to fit different aspects of human data such as the effect of SAT and difficulty manipulations on accuracy, RT and confidence. We found that RTNet fits all these aspect of the data including the shape and skewness of RT distributions. However, one of the main aspects of the data RTNet has trouble fitting well is the stochasticity of decisions. When presented with the same stimulus multiple times, humans tend to make more inconsistent choices than RTNet. Since this finding is not related to confidence, it does not seem relevant to discuss in this paper. However, we now acknowledge this limitation in the Discussion and paste the relevant section below:

A limitation of using RTNet as a model of human perceptual decisions is that RTNet underestimates the stochasticity of human decisions. When presented with the same stimulus multiple times, humans tend to make more inconsistent choices than RTNet. However, in spite of this limitation, RTNet’s RTs and confidence correlated significantly with those of individual subjects (Rafiei et al., 2024), suggesting that RTNet can still function as sufficiently a good model of human choice and RT to allow meaningful comparison of confidence strategies.

***Clarification:
I think it would be useful for readability to have some more description of the model architectures and training the main Results.***

We agree and have now included additional description of the RTNet model architecture. Note that due to the fact that we have now moved the remaining architectures to the Supplementary, we describe the details of those architectures also in the Supplementary. We paste the relevant sections from the Results below:

Each strategy was implemented within our recently developed neural network model, RTNet (Rafiei et al., 2024). The RTNet architecture consists of two modules – (1) a Bayesian neural network (BNN) with stochastic weights and (2) an evidence accumulator. The stochastic weights of the BNN generate a unique feedforward network at each time step and lead to variable activations in the network’s final layer. The evidence accumulator receives these noisy activations from the BNN and integrates the evidence towards a pre-defined threshold. Evidence is accumulated independently for each choice option and the model’s decision corresponds to the option for which the evidence first crosses the threshold.

In our previous work, we had trained RTNet on the MNIST dataset and then fit the noise and boundary parameters (see Methods) to match human performance for difficulty and speed-accuracy trade-off manipulations (Rafiei et al., 2024). Here, we fit the same pre-trained RTNet model to the human confidence ratings using the activations at the final time-step when the model generated the decision. Specifically, for each confidence strategy, we fit a set of three confidence criteria that transform the continuous confidence decision variable for each confidence strategy into 4-point confidence ratings. We fit each model to the data of each human subject separately. We then assessed the quality of model fits and the predictions of each strategy for the confidence associated with previously unseen individual images.

***Please add clarification to the methods about what timepoint/layer the logits/raw activations are taken from for confidence calculations. (I'm assuming it is the timepoint where the model reached the threshold or the layer they did, depending on model architecture).***

The logits and raw activations are indeed taken from the timepoint at which the model reached the threshold. We have now added additional clarification about the methods and paste the relevant section below:

Confidence is computed from the evidence at the final time step at which the network makes a decision. We implemented seven confidence strategies: PE, Top2Diff, Softmax, BCH, ProbTop2Diff, ProbAvgRes, and Entropy. We first obtained the accumulated evidence, that is, the logits or raw activations for each of the eight choice options, z=[z_1,z_2,…z_8].

***typo: "Second, we evaluated the effect of the speed-accuracy trade-off manipulation on
confidenceClick or tap here to enter text.."***

We thank the reviewer for catching this typo. We have corrected it.

**Reviewer #2**

***The current report examines the best way to compute confidence in deep neural networks, in order to mimic human confidence judgments. From a technical perspective this excellent work, and likewise the manuscript is very well written. From a conceptual standpoint, however, the novel insights are less clear to me. Most researchers interested in human confidence would not be surprised that softmax is not a good rule to capture human confidence (I’m not aware of any theory making such claims) and likewise most researchers working with DNNs would not be interested in whether they model human confidence (but instead would want the most informative measure).***

We thank the reviewer for the comments. We agree that it is important to ensure that our study makes a significant conceptual advance, apart from replicating and extending findings from previous studies. In order to address the reviewer’s concern, we have now included three other confidence strategies. Specifically, we test three new hypotheses regarding how confidence is computed – 1) confidence as the posterior probability of the choice (the Bayesian confidence hypothesis or BCH; Aitchison et al., 2015), 2) confidence as a difference in posterior probability between the top-two choices (ProbTop2Diff; Li & Ma, 2020), and 3) confidence as a difference in probability between the choice and the average across the remaining choices (ProbAvgRes; Comay et al., 2023; as per the reviewer’s request). To obtain posterior probabilities, we train a separate metacognition module that learns the distributions of likelihood across choices. We also modify our Entropy model to now use these posterior probabilities for its computations instead of the softmax probabilities.

These new models allow us to compare aspects of confidence computations that have not been tested before for multi-alternative tasks. For instance, not only can we test whether confidence is computed from all available information or from different kinds of subsets, but now we can directly test whether Bayesian computations are necessary for generating confidence. Further, there is also an open debate on whether confidence relies on probabilities or on raw evidence. Comparisons between Top2Diff and ProbTop2Diff can now allow us to inform this debate. We believe that these new models now provide novel insights into confidence computations for multi-alternative tasks. We explain these findings in more detail below.

***1. For metacognition researchers the conclusion that human confidence is not well explained by softmax or entropy is not very surprising. First, it is the same conclusion reached by Li & Ma, admittedly using a more complex model that works with actual images. Also, I am not aware of any theory of confidence arguing that humans compute confidence using softmaxt or entropy?***

We agree with the reviewer that the conclusion about Softmax and Entropy is not very surprising since Li and Ma (2020) also show that the Entropy strategy fails to provide good fits to human data. Nevertheless, since these strategies have not been explicitly tested for an 8-choice task or within artificial neural network models, it is not a priori clear that these strategies will fail to perform. Therefore, for the sake of completeness, we include the Softmax and Entropy models in this study.

More importantly, however, in Li and Ma’s study, the best fitting model was found to be one that computes confidence as the *difference in posterior probabilities* between the top-two choices. On the other hand, the best fitting model in our study was one that computed the difference in *raw evidence* between the top two choices. Therefore, it is still unclear which version of the Top2Diff model fits human data better. We now find that the Top2Diff model based on raw evidence (our original model) outperforms the Li & Ma model based on difference in posterior probabilities (which we refer to as ProbTop2Diff here). These findings allow us to conclude not only that confidence uses only a subset of the available information but also that it relies on simple computations on raw evidence rather than complex probability calculations. We have made extensive edits throughout the paper to address these new models and the new results obtained with them. We paste all sections that have been substantially altered:

**Abstract:**

Humans can evaluate the accuracy of their decisions by providing confidence judgements. Traditional cognitive models have tried to capture the mechanisms underlying this process but remain mostly limited to two-choice tasks with simple stimuli. How confidence is given for naturalistic stimuli with multiple choice alternatives is not well understood. We recently developed a convolutional neural network (CNN) model – RTNet – that exhibits several important signatures of human decision making and outperforms existing alternatives in predicting human responses. Here, we use RTNet’s image computability to test seven different confidence strategies on a task involving digit discrimination with eight alternatives (N=60). Specifically, we compared confidence strategies that consider the entire evidence distribution across choices against strategies that only consider a specific subset of the available evidence. We also compared strategies based on whether they compute confidence from posterior probabilities or from raw evidence. A model which derives confidence from the difference in raw evidence between the top-two choices (Top2Diff model) consistently provided the best quantitative and qualitative fits to the data and the best predictions of human confidence. These results support the notion that human confidence is based on a specific subset of available evidence, challenge prominent theories such as the Bayesian confidence hypothesis and the positive evidence heuristic, and establish CNNs as promising models for inferring the mechanisms underlying human confidence in naturalistic settings.

**Introduction:**

The three strategies mentioned above derive confidence directly from the evidence underlying the choice. However, another popular set of theories is based on the assumption that confidence relies on explicit probability computations rather than raw evidence. For instance, confidence can be computed as the Entropy of the posterior probability distribution across all available choices (Entropy strategy; Li & Ma, 2020; Spoerer et al., 2020). Alternatively, Comay et al. (2023) proposed that confidence is computed as the difference between the posterior probability of the most likely choice and the average of posterior probabilities of the residual choices (the ProbAvgRes model). In contrast, other strategies use only a subset of the posterior distribution. Li & Ma (2020) implemented a probability-based version of the Top2Diff model where confidence is computed as the difference in posterior probabilities of the two most likely choices (the ProbTop2Diff). Finally, the Bayesian confidence hypothesis (the BCH model), one of the most popular theories in the field, proposes that confidence is the posterior probability associated with the chosen response (Hangya et al., 2016; Sanders et al., 2016). Confidence in the BCH model uses the posterior probability associated with just the chosen category, and unlike the remaining probability-based models above, ignores the probability associated with all other choices.

Despite the fact that various confidence strategies have been proposed, there is currently little empirical work to distinguish between them. Perhaps the only attempts to quantitatively compare some of these strategies in the context of a multi-alternative task were made by Li & Ma (2020) and Comay et al. (2023). Li & Ma (2020) applied Bayesian modelling to a 3-choice task and showed that the ProbTop2Diff model outperformed both the BCH and Entropy models. However, Li & Ma’s task was unique in that, unlike most of the literature, none of the alternatives were objectively correct on any given trial. Comay et al. (2023) used a similar approach to model confidence but for a three-choice task where there was indeed an objectively correct answer on every trial. Their results showed that ProbAvgRes outperformed BCH and ProbTop2Diff. However, neither of these studies examined evidence-based models, despite recent evidence that confidence computations are likely performed on the raw sensory evidence (Xue et al., 2023) Further, both of these studies used relatively simple stimuli, and both only examined three-choice tasks, leaving it unclear how confidence is computed in cases with higher number of alternatives and complex images.

**Results:**

Quantitative model comparisons

We first compared how well the seven confidence strategies fit the observed data using AIC scores. Lower AIC scores indicate better fits to the data. AIC comparisons revealed that the Top2Diff strategy significantly outperformed all other strategies by generating the lowest AIC values (**Figure 2A**). Specifically, Top2Diff outperformed the ProbAvgRes strategy by an average of 11.64 points (95% CI = [4.26, 18.19]), the ProbTop2Diff strategy by 15.66 points (95% CI = [7.57, 24.24]), the Entropy strategy by 22.43 points (95% CI = [11.69, 35.95]), the PE strategy by 58.68 points (95% CI = [46.74, 76.74]), the Softmax strategy by 69.79 points (95% CI = [51.28, 103.34]), and the BCH strategy by 80.59 (95% CI = [60.61, 108.77]) points. These differences correspond to the Top2Diff strategy being, on average, 337 times more likely than ProbAvgRes, 2521 times more likely than ProbTop2Diff, 7.4 x 10^4^ times more likely than Entropy, 5.3 x 10^12^ times more likely than PE, 1.4 x 10^15^ times more likely than Softmax, and 3.2 x 10^17^ times more likely than BCH. In other words, the Top2Diff strategy provided a much better quantitative fit to the human data compared to any of the other strategies.

***Figure 2. Comparing the ability of confidence strategies to fit human confidence ratings.*** *We examined the quantitative fits of the seven confidence strategies (PE, BCH, Top2Diff, ProbTop2Diff, ProbAvgRes, Entropy and Softmax). The Top2Diff strategy outperformed all other confidence strategies by an average of at least 11 AIC points. Error bars depict 95% confidence intervals for the difference in AIC scores between Top2Diff and each of the remaining strategies.*

Qualitative model comparisons

To gain further insight into the performance of the seven confidence strategies, we compared how well each strategy can fit different qualitative patterns of confidence. Specifically, we examined the effects of the two behavioral manipulations (task difficulty and speed-accuracy trade-off) on confidence. In addition, we examined the folded-X pattern which is popularly regarded as a critical signature of human confidence (Hangya et al., 2016; Sanders et al., 2016).

First, we evaluated the effect of task difficulty manipulation on confidence. For humans, we found that confidence decreased from 3.50 in the easy to 3.14 in the difficult condition (t(59) = 11.768, p < 0.001; **Figure 3A**). All strategies could replicate this general pattern (Top2Diff: easy = 3.49, difficult = 3.26; ProbAvgRes: easy = 3.50, difficult = 3.22; ProbTop2Diff: easy = 3.50, difficult = 3.24; Entropy: easy = 3.47, difficult = 3.28; PE: easy = 3.41, difficult = 3.31; Softmax: easy = 3.50, difficult = 3.28; BCH: easy = 3.54, difficult = 3.27). Numerically, the ProbTop2Diff strategy produced the smallest error magnitude as measured by the average sum of squared errors across subjects (Top2Diff = 0.041; ProbAvgRes = 0.040; ProbTop2Diff = 0.037; Entropy = 0.050; PE = 0.070; Softmax = 0.094; BCH = 0.077). However, these errors were not significantly different from the errors generated by the Top2Diff and ProbAvgRes models (Top2Diff: t(59) = 1.094, p = 0.28; ProbAvgRes: t(59) = 0.617, p = 0.54). The remaining strategies – Entropy, PE, Softmax and BCH produced significantly worse fits compared to ProbTop2Diff (Entropy: t(59) = 2.278, p = 0.026; PE: t(59) = 4.974, p < 0.001; Softmax: t(59) = 3.342, p = 0.001; BCH: t(59) = 3.415, p = 0.019).

***Figure 3. Comparing the ability of confidence strategies to fit patterns of human confidence.*** *We examined the qualitative fits of the seven confidence strategies. (A) Confidence decreases with task difficulty. All confidence strategies were able to reproduce this qualitative pattern, but the Top2Diff, ProbAvgRes and ProbTop2Diff models provided the closest fits to the data. (B) There was no significant change in confidence between the speed and accuracy focus conditions. All models incorrectly predicted that confidence should be higher in the accuracy- compared to the speed-focus condition, but ProbAvgRes and ProbTop2Diff models provided the closest fits to the data. (C) Confidence increased with task performance for correct trials but decreased with task performance for error trials giving rise to a folded-X pattern. All strategies except Entropy can reproduce this qualitative pattern, but the Top2Diff, ProbAvgRes and ProbTop2Diff models provided the closest fits to the data. Error bars depict SEM. SSE, sum of squared errors (smaller values indicate better fits).*

Second, we evaluated the effect of the speed-accuracy trade-off manipulation on confidence. In humans, there was no significant change in confidence from the speed-focus to the accuracy-focus condition (speed-focus = 3.31, accuracy-focus = 3.33; t(59) = 0.612, p = 0.543; **Figure 3B**), a somewhat unusual finding since other research has consistently found an increase in confidence from the speed-focus to the accuracy-focus conditions (Pleskac & Busemeyer, 2010; Rahnev et al., 2016). Unlike the data in the current study but consistent with the previous literature, all models showed an increase in confidence from the speed-focus to accuracy-focus condition (Top2Diff: 3.16 vs. 3.58; ProbAvgRes: 3.22 vs. 3.50; ProbTop2Diff: 3.22 vs. 3.52; Entropy: 3.06 vs. 3.68; PE: 2.94 vs. 3.77; Softmax: 3.11 vs. 3.68; BCH: 3.29 vs. 3.51). Computing the average sum of squared errors across subjects showed that the ProbAvgRes model provided numerically the closest match to human confidence (Top2Diff = 0.155; ProbAvgRes = 0.121; ProbTop2Diff = 0.121; Entropy = 0.272; PE = 0.459; Softmax = 0.354; BCH = 0.142). These errors were significantly lower for the ProbAvgRes model compared to all other models except ProbTop2Diff and BCH (Top2Diff: t(59) = 5.170, p < .001; ProbTop2Diff: t(59) = 0.018, p = 0.986; Entropy: t(59) = 10.280, p < 0.001; PE: t(59) = 9.730, p < 0.001; Softmax: t(59) = 5.522, p < 0.001; BCH: t(59) = 1.521, p = 0.134).

Next, we assessed whether our human subjects and models generated the “folded-X pattern” (Hangya et al., 2016; Sanders et al., 2016), where, as the task gets easier, confidence for correct trials increases but confidence for error trials decreases. Indeed, human confidence followed this folded-X pattern, such that the easy condition exhibited higher confidence for correct trials (difficult = 3.39, easy = 3.66; t(59) = 9.727, p < 0.001; **Figure 3C**) but lower confidence for error trials (difficult = 2.74, easy = 2.68; t(59) = -2.095, p = 0.041). All strategies predicted that confidence for correct trials should be higher for the easy compared to the difficult condition (Top2Diff: difficult = 3.46, easy = 3.62; ProbAvgRes: difficult = 3.44, easy = 3.61; ProbTop2Diff: difficult = 3.44, easy = 3.61; Entropy: difficult = 3.43, easy = 3.56; PE: difficult = 3.40, easy = 3.47; Softmax: difficult = 3.46, easy = 3.60; BCH: difficult = 3.48, easy = 3.65). Similarly, all models correctly predicted that confidence for error trials should be lower for the easy compared to the difficult condition (Top2Diff: difficult = 2.96, easy = 2.82; ProbAvgRes: difficult = 2.91, easy = 2.87; ProbTop2Diff: difficult = 2.94, easy = 2.88; Entropy: difficult = 3.05, easy = 2.90; PE: difficult = 3.16, easy = 3.09; Softmax: difficult = 3.01, easy = 3.00; BCH: difficult = 3.95, easy = 3.92). Numerically, the ProbAvgRes model produced the most precise fits to the folded-X pattern observed in humans as measured by the sum of squared errors across subjects (Top2Diff = 0.245; ProbAvgRes = 0.238; ProbTop2Diff = 0.291; Entropy = 0.460; PE = 0.558; Softmax = 0.437; BCH = 0.308). However, these errors were not significantly different compared to the Top2Diff and ProbTop2Diff models (Top2Diff: t(59) = 0.433, p = 0.66; Top2Diff: t(59) = 1.822, p = 0.07) but were significantly lower compared to all other models (Entropy: t(59) = 3.437, p = 0.001; PE: t(59) = 8.165, p < 0.001; Softmax: t(59) = 3.482, p < 0.001; BCH: t(59) = 2.113, p = 0.038).

Overall, as may be expected from the AIC fits, the top three models which yielded the best AIC fits – Top2Diff, ProbAvgDiff, ProbTop2Diff – produced the most consistently accurate matches to the observed patterns of human confidence across all three qualitative patterns.

Assessing model predictions of confidence for stimulus categories

Apart from capturing the behavioral patterns of different experimental manipulations, a robust model of confidence should also account for stimulus-driven variations. Specifically, certain categories of stimuli (such as the number 1) may be more prone to confusion with other digits (such as 7) and therefore, may be associated with lower confidence on average. Therefore, we assessed whether models could mimic the observed patterns of confidence across the eight stimulus categories. In addition to stimulus categories, we separated trials based on choice accuracy, since observed patterns of confidence depend on the accuracy of the primary choice. Confidence on error trials is generally lower compared to correct trials and confidence for correct and error trials have opposing relationships with stimulus discriminability (**Figure 3C**). Mixing confidence across correct and error trials can thus obscure the underlying patterns.

First, we computed average confidence as a function of choice accuracy and stimulus category. Although confidence was generally well matched across the eight stimulus categories, there were small variations between categories. For correct trials, humans tended of give the highest confidence for the digit 8, and the lowest confidence for the digit 1. For error trials, the digit 8 was again associated with the highest confidence while 6 was associated with the lowest confidence. As expected, confidence was always higher for correct trials compared to error trials for humans as well as all models across all stimulus categories (**Figure 4**). Model comparisons showed that the Top2Diff model gave the closest fits to the overall pattern of confidence as assessed by the sum of squared errors (Top2Diff = 1.391; ProbAvgRes = 2.100; ProbTop2Diff = 2.035; Entropy = 2.427; PE = 3.402; Softmax = 2.088; BCH = 2.231). These errors were significantly lower for the Top2Diff model compared to all other models (ProbAvgRes: t(59) = 7.970, p < .001; ProbTop2Diff: t(59) = 6.124, p < 0.001; Entropy: t(59) = 4.141, p < 0.001; PE: t(59) = 9.922, p < 0.001; Softmax: t(59) = 3.169, p = 0.002; BCH: t(59) = 6.780, p < 0 .001). In particular, for error trials, all other models produce substantial deviations from observed confidence for at least two stimulus categories, with the PE, Softmax, and Entropy models consistently overpredicting confidence across all stimulus categories.

***Figure 4. Stimulus category-wise predictions of average confidence for correct and error trials.*** *Confidence was uniformly greater for correct trials compared to error trials across all eight stimulus categories. In addition, there were small variations in confidence across the eight categories, possibly due to some digits being more prone to confusion with others. All confidence strategies correctly predicted that confidence was higher for correct trials compared to error trials, but Top2Diff best captured the variations in confidence found between the stimulus categories. In particular, all models except Top2Diff over-estimated confidence for error trials for at least two categories, with the PE, Softmax, and Entropy models over-estimating confidence for all stimulus categories. Error bars depict SEM. SSE, sum of squared errors (smaller values indicate better fits).*

Second, we analyzed how well models predicted the variations in confidence across stimulus categories for individual subjects. For each subject, we plotted averaged confidence within each stimulus category against the model’s predicted confidence for that stimulus category, separately for correct and error trials. For correct trials (**Figure 5A**), all confidence strategies were able to predict individual confidence within the stimulus categories well – with predicted confidence falling within the observed range and showing a continuous increase within the range. None of the models showed a substantial tendency to over- or under-estimate confidence. However, for error trials (**Figure 6A**) the Entropy, PE, and Softmax models uniformly over-estimated confidence across stimulus categories as well as individuals, which may explain their poor fits, compared to the top-three models – Top2Diff, ProbAvgRes and ProbTop2Diff.

***Figure 5. Stimulus category-wise model predictions of individual confidence for correct choices.*** *(A) We computed average confidence for correct trials within each stimulus category separately for each individual subject and plotted these against the corresponding quantities predicted by each model. All models generated strong and significant correlations with observed confidence, suggesting that they were able to capture stimulus-related variations in confidence. (B) The Top2Diff strategy generated the numerically highest correlations with human confidence. However, these correlations were not significantly higher than those generated by ProbAvgRes, ProbTop2Diff, Entropy, and PE models. Error bars show 95% confidence intervals. (C) We fit a linear-mixed model that quantified how closely each model captured observed category-wise variations in confidence, while controlling for the repeated measurement across individuals. AIC scores derived from the linear mixed models showed that the Top2Diff strategy generated the best fits to category-specific confidence for correct trials with an AIC difference of at least 10 points with all other strategies.*

***Figure 6. Stimulus category-wise model predictions of individual confidence for error trials.*** *(A) We computed average confidence for error trials within each stimulus category separately for each individual subject and plotted these against the corresponding quantities predicted by each model. As with correct trials, models generated strong and significant correlations with observed confidence, suggesting that they are generally able to capture stimulus-related variations in confidence. (B) The Top2Diff strategy generated the numerically highest correlations with human confidence, but these correlations were not significantly higher than those generated by ProbAvgRes, ProbTop2Diff, PE and BCH models. Error bars show 95% confidence intervals. (C) We fit a linear-mixed model that quantified how closely each model captured observed category-wise variations in confidence, while controlling for the repeated measurement across individuals. AIC scores from these regression models showed that the Top2Diff strategy generated the substantially better fits to category-specific confidence for error trials with an AIC difference of at least 62 points with all other strategies.*

To quantify how well models could predict the variations in confidence between stimulus categories, we fit a linear mixed model which predicted human confidence for each stimulus category from the model’s confidence for that category. Since the category-wise predictions of confidence were obtained separately for each individual subject, we controlled for the repeated measurements of these stimuli by including stimulus category as a random effect in the model. Using the mixed linear model’s estimates of slope and intercept, we generated the model’s predictions of category-wise confidence across individuals and correlated them with their corresponding observed quantities.

For correct trials, all strategies produced very strong correlations with human confidence (all r’s > 0.871, all p’s < 0.001; **Figure 5B**) across the eight stimulus categories. Comparing the Pearson’s r-values showed that the Top2Diff model generated the numerically highest correlations, which were significantly higher than those produced by the Softmax and BCH models (Softmax: z(479) = 3.789, p < 0.001; BCH: z(479) = 3.329, p < 0.001). However, the Top2Diff model’s correlations were not significantly higher than the ProbAvgRes, ProbTop2Diff, Entropy, and PE models (ProbAvgRes: z(479) = .939, p = 0.348; ProbTop2Diff: z(479) = .196, p = .844; Entropy: z(479) = .196, p = .844; PE: z(479) = .576, p = .565). In addition to these correlations, we also quantified the goodness-of-fit of the linear mixed model using AIC scores and found that the Top2Diff strategy generated the closest predictions of category-wise confidence for correct trials across individual subjects, with a difference of at least 10 AIC points with all other strategies (**Figure 5C**).

For error trials, the category-wise correlations between model and human confidence were slightly lower but nevertheless strong and significant for all confidence strategies (all r’s > 0.818, all p’s < 0.001; **Figure 6B**). Comparing the Pearson’s r-values showed that the Top2Diff model once again generated the numerically highest correlations, which were significantly higher than those produced by the Entropy and Softmax models (Entropy: z(479) = 2.100, p = 0.036; Softmax: z(479) = 2.627, p = 0.009). However, the Top2Diff model’s correlations were not significantly higher than those produced by the ProbAvgRes, ProbTop2Diff, PE, and BCH models (ProbAvgRes: z(479) = 1.540, p = .124; ProbTop2Diff: z(479) = 1.644, p = .100; PE: z(479) = .1.110, p = .267; BCH: z(479) = .944, p = .345). Comparison of AIC scores from the linear mixed model showed that the Top2Diff strategy also generated the best fits to category-wise confidence for error trials across individual subjects, with a substantial difference of at least 62 AIC points with all other strategies (**Figure 6C**). Together, these results suggest that the Top2Diff strategy best captures the patterns of variations in human confidence arising from dependencies in the stimulus categories.

**Discussion:**

Our results support the view that human confidence judgments are unlikely to reflect complex probability computations (Adler & Ma, 2018b, 2018a; Denison et al., 2018; Li & Ma, 2020; Rausch et al., 2020; Shekhar & Rahnev, 2024a; Xue et al., 2023 ). Previous studies on confidence in multi-alternative perceptual tasks only compared probability-based models (Comay et al., 2023; Li & Ma, 2020). However, recent research shows that probability-based models rarely provide a good description of human confidence (Locke et al., 2021). For example, several previous studies have strongly challenged the assumption that confidence follows the BCH strategy, which is the most prominent probability-based model (Adler & Ma, 2018b, 2018a; Denison et al., 2018; Li & Ma, 2020 ). It is therefore critical for future studies of confidence to always consider models where confidence is based directly on the sensory evidence and not solely focus on probability-based confidence models.

***Second, a recent paper by Comay et al. 2023 (Cognition) showed tat confidence depends on a dud-alternative and proposed a average-residual account for their findings. It would be important to also test this strategy, and perhaps see whether the top2 rule can account for the dud effect (given that this seems to directly contradict predictions from this rule).***

We thank the reviewer for this suggestion and have now implemented this confidence strategy. However, it should be noted that the design of our task does not allow us to test for the dud-alternative effect since we only present a single stimulus on each trial.

***2. The authors model confidence based on the (accumulated) activation in the output layer, and call this evidence. I have some concerns about this. First, the top2 rule seems to imply that humans do not consider the evidence of other options, but this conclusion is only valid if the nodes in the output layer are independent. If that is not the case, and especially for MNIST it is not hard to conceive that evidence for 8 and 9 will not be independent from each other, the top2 rule could be an indirect way to consider all evidence.***

This is an important distinction. The Top2Diff model says that confidence is based only on the top-2 alternatives in the final layer. However, the reviewer is correct to note that the evidence for different alternatives is likely correlated. In that sense, knowing the evidence for even just two alternatives already means that one has partial knowledge about the likely evidence for the other alternatives. Correspondingly, we don’t claim that information about the other alternatives is completely lost. It’s just that the computation itself does not explicitly consider the evidence for those other alternatives. We now clarify this point about the Top2Diff model in the Discussion:

It is important to note that the Top2Diff strategy does not imply that information about the other alternatives is completely lost since the evidence generated for all the choice alternatives is driven by the same stimulus and likely to be correlated. In that sense, knowing the evidence for even just two alternatives means that one has partial knowledge about the likely evidence for the other alternatives. The Top2Diff model simply assumes that the computation itself does not explicitly consider the evidence for those other alternatives.

***Second, I wonder whether this is the most sensible way to think about evidence, for example compared to a metacognitive module having access to the full processing stream of evidence. Why is confidence only calculated based on the final output node?***

The reviewer suggests an interesting possibility that confidence may be based on the entire stream of evidence rather than on the evidence in the final output layer. However, deriving confidence from all the nodes of the network is a highly complex computation that may be practically unfeasible. For instance, there are thousands of nodes even within a single layer of the network and it is unclear how these nodes relate to the evidence for a given option. Further, these networks are structured in a way that the final layer is the one that is maximally informative about the evidence for each option and considering the previous layers may only add noise to the computation. We now discuss this issue in the Methods and paste the relevant section below:

In all these computations, it is assumed that confidence arises from the final processing layer of RTNet. It is in principle possible to derive confidence using information in all layers of RTNet rather than the information in the last layer only. However, implementing this possibility would require one to interpret evidence from all the nodes of a neural network, which is a highly complex computation that may be practically unfeasible. For instance, there are thousands of nodes even within a single layer of the network and it is unclear how these nodes relate to the evidence for a given option. Further, these networks are structured in a way that the final layer is the one that is maximally informative about the evidence for each option and considering the previous layers may only add noise to the computation. Therefore, for these neural network models, we assume that confidence is derived entirely from the evidence in the final layer of the network.

Nevertheless, we now partially implement the reviewer’s suggestion of having a separate metacognition module which is trained on the output of the network. We use this metacognition module to compute confidence using the posterior probability distribution across choices for 4 of the models examined here – the Bayesian Confidence Hypothesis (BCH model), the difference in posterior probabilities between the top-two options (ProbTop2Diff model from Li & Ma, 2020), the difference in probabilities between the best option and the average of all other options (ProbAvgRes model from Comay et al., 2023), and the entropy of the posterior distribution (Entropy model).

***3. It is a bit worrisome that one of the three key empirical findings in the human data are not captured by (any of) the model(s). Humans do not give higher confidence in accuracy vs speed conditions, yet all models predict this. This is a rather glaring misfit that the authors seem to dismiss rather easily. Does this not point to a clear issue with the model(s)?***

We agree with the reviewer that it is important to discuss the mismatch between the model predictions and data. In this case, we believe that the mismatch is due to the data being under-powered rather than due to a failure of the models. We looked at existing studies that investigated the effects of the speed-accuracy trade-off on confidence and found that across three different experiments, confidence always increased from the speed-focus to the accuracy-focus conditions (Pleskac & Busemeyer, 2010; Rahnev et al., 2016). In our study, it is likely that we failed to find a significant increase in confidence from the speed to accuracy-focused conditions due to a small effect size, which could be due to the complexity of the task (8-choices). In fact, for the trials within the difficult (high noise) condition, the increase in accuracy itself between the speed and accuracy conditions did not reach significance (p = 0.186). Since changes in confidence are likely to be driven by underlying changes in accuracy, these weak overall difference in accuracy between the speed and accuracy conditions may be responsible for the absence of a significant effect of SAT on confidence in the human data. Overall, we feel that it’s the human data on confidence that is slightly unusual here and that the models’ behavior is as expected. Nevertheless, we agree with the reviewer that this mismatch has to be discussed more prominently. Therefore, we now discuss this as a limitation of our study in the Results and Discussion, and paste the relevant sections below:

A final limitation of our study is that the complexity of the task may have made it difficult to realize the speed-accuracy trade-off manipulation. Our task structure was relatively complex – featuring eight choice options and a 2 x 2 factorial design with difficulty and SAT manipulations. Such a complex task structure may have reduced the effect of the SAT manipulation. Indeed, the SAT manipulation had a very small effect on accuracy (2-3%; significant for the easy stimuli but not for the difficult stimuli), making it less surprising that the effect on confidence was also very small (and not significant). Previous studies, on the other hand, have reported an increase in confidence from the speed-focus to the accuracy-focus conditions (Pleskac & Busemeyer, 2010; Rahnev et al., 2016), suggesting that increases in confidence do indeed occur with SAT manipulations. All confidence strategies examined here led to an increase of confidence from the speed-focus to the accuracy-focus conditions. Given the considerations above, we believe that this divergence from the current data should not be seen as a limitation of the models but as a limitation of the current dataset.

***4. One of the first papers examining human confidence in DNNs, by Webb and colleagues (2023) is only briefly mentioned in the discussion as a paper implementing softmax confidence. My reading from this paper, however, is that confidence is implemented as a separate head which learns to compute confidence via reinforcement learning. As such, we don’t really know whether or not it learned to use softmax, or whether perhaps it learned to use the top2 diff rule. Likewise in the discussion Rafiei et al. (2024) is dismissed as using the softmax rule, but in that paper it reads “The confidence of the model was obtained by taking the difference in evidence scores between the chosen response and the second-best choice.” Is this not the top2 rule?***

We apologize for the confusion and want to clarify that in Rafiei et al. (2024), the RTNet model indeed computed confidence as the difference in evidence between the top two choices. Secondly, in Webb et al (2023), their main model learnt to implement confidence through reinforcement learning as the probability of the choice. However, in their supplementary, they test another version of their network where confidence was computed using the softmax function. To avoid any confusion, we have now removed both these citations and cite only those papers which originally computed confidence from the softmax function.

***5. As a minor point: it would be interesting to get some insights about how all of this work relates to related human confidence literature. For example, is the top 2 rule consistent with confidence as a distance-to-criterion signal?***

We thank the reviewer for the suggestion. The addition of the three new models which compute confidence from the posterior probability distribution across choices now gives us more insight into whether confidence is based on probability computations or raw evidence. We find that confidence is more likely to be based on raw evidence i.e. the Top2Diff rule rather than on probability computations, which is consistent with previous findings in the literature for two-choice tasks with simple stimuli (Xue et al., 2024). We discuss these new insights throughout the updated paper.
